# Challenges in Expanding Access to the HPV Vaccine Among Schooling Girls: A Mixed-Methods Study from Indonesia

**DOI:** 10.3390/vaccines13090948

**Published:** 2025-09-04

**Authors:** Jacqueline Yap, Fauzi Budi Satria, Ivana Alona, Indo Mora Siregar, Shu Chen, Chee Fu Yung, Courtney Davis, Inke Nadia Diniyanti Lubis, Shenglan Tang

**Affiliations:** 1 Department of Biochemistry, Duke-NUS Medical School, Singapore 169857, Singapore; 2 Faculty of Medicine, Universitas Sumatera Utara, Medan 20222, Indonesiaivana@usu.ac.id (I.A.);; 3 Australia National Centre for Immunisation Research and Surveillance, Westmead 2145, Australia; 4 Department of Population Health Science, Duke Global Health Institute, Kunshan 215316, China; 5 KK Women’s and Children’s Hospital, Singapore 229899, Singapore; 6 SingHealth Duke-NUS Paediatric Academic Clinical Programme, Duke-NUS Medical School, Singapore 169857, Singapore; 7 SingHealth Duke-NUS Global Health Institute, Duke-NUS Medical School, Singapore 169857, Singapore

**Keywords:** human papillomavirus vaccines, vaccine coverage, influencing factors

## Abstract

Background: Indonesia launched a nationwide school-based HPV immunization program in August 2023. Despite this, regional disparities in vaccine uptake persist. Therefore, we undertook a study in North Sumatra Province to assess HPV vaccination coverage and analyze the main factors affecting the uptake of HPV vaccination. Methods: This study employed a mixed-methods approach and was carried out in Medan and Deli Serdang of North Sumatra Province. Quantitative data were used to examine HPV coverage rates among school-aged girls in 2024, while qualitative interviews with parents, teachers, and health officers explored administrative, social, and behavioral barriers and facilitators. Results: In 2024, HPV vaccine coverage in Deli Serdang reached 62.09%, while Kota Medan lagged behind at just 27.20%. High-coverage schools in the Galang subdistrict benefited from proactive engagement between Puskesmas (community health clinics) and parents. In contrast, lower-coverage areas experienced logistical and communication challenges. Parents expressed a preference for face-to-face communication over written consent forms and emphasized the importance of clear, empathetic messaging. Conclusions: The stark contrast in coverage—particularly the low uptake in urban Kota Medan—highlights the need for more responsive and localized implementation strategies. Strengthening direct communication, addressing administrative inefficiencies, and fostering trust through tailored community engagement are critical. These findings suggest a need for targeted improvements in urban settings and further research across diverse regions to inform policy development and strategies for improved coverage of HPV vaccinations.

## 1. Introduction

Human papillomavirus (HPV) is associated with cervical and other genital cancers. With persistent infection, HPV can lead to cervical intraepithelial neoplasia (CIN) and development of cancer. In 2022, low- and middle-income countries (LMICs) accounted for approximately 94% of the 350,000 cervical cancer-related deaths [1].

In Indonesia, a 2018 study estimated that cervical cancer mortality led to 246,350 years of life lost (YLL), translating to a productivity loss of approximately IDR 23.174 trillion (USD 1.6 billion) [2]. In 2018, the World Health Organization (WHO) Director-General issued a global call to eliminate cervical cancer, leading to the adoption of the Global Strategy for Cervical Cancer Elimination by the World Health Assembly in 2020 [3]. Globally, 115 countries have incorporated HPV vaccination into their national immunization programs, including 51 high-income countries (44.35%) and 64 low- and middle-income countries (55.65%) [4].

Indonesia is an archipelago in Southeast Asia consisting of approximately 17,000 islands and is the fourth most populous country in the world. As of 2023, it has a Gross Domestic Product (GDP) per capita of USD 4940.55, and the health expenditure accounts for 3.71% of its GDP in 2021 [5]. It has graduated from Gavi but still qualifies under the Gavi middle-income countries (MICs) support. Indonesia’s HPV immunization program was initially introduced between 2016 and 2021 in 20 districts and cities before expanding to 112 additional districts by 2022. The nationwide scale-up of HPV vaccines has been school-based, with a focus during National Child Immunization Month, known as Bulan Imunisasi Anak Sekolah (BIAS), which is a routine activity to provide immunizations for primary school-aged children both attending and not attending school [6]. In August 2023, the program was officially launched nationwide, primarily targeting girls in grades 5 and 6 (ages 11–12) through school-based vaccinations. The HPV elimination plan, structured in three phases, aims to achieve 90% vaccination coverage among school-going girls aged 11–12 by 2027 [7].

Indonesia produces Nusagard domestically, a four-valent HPV vaccine, through a collaboration between MSD and Bio Farma, since 2023 [8]. To enhance vaccine management, the Ministry of Health, with United Nations Development Programme (UNDP) support, developed Sistem Monitoring Informasi Logistik secara Elektronik (SMILE) (Kota Batam, Indonesia)—a mobile and web-based system for real-time monitoring of cold chain logistics, including stock management and temperature tracking [9]. Additionally, ASIK (Sehat IndonesiaKu) (Paris, France), launched in May 2022, is a digital health tool for tracking vaccinations and health services. Used by healthcare workers at Public Health Centers, or Pusat Kesehatan Masyarakat (Puskesmas), it records individual health data, enabling service documentation, follow-ups, and patient monitoring [10].

According to modeling estimates by WHO and UNICEF, Indonesia’s national HPV immunization coverage was just 3% in 2020, rising modestly to 6% by 2022 [11]. This low coverage was also reflected in a regional assessment by the Asian National Cancer Centers Alliance (ANCCA), which similarly reported a 6% HPV vaccination rate for Indonesia in 2022 [12]. However, with the national rollout of the HPV vaccination program in 2023, coverage rates have since increased significantly. Data from the WHO/UNICEF Joint Reporting Form on Immunization, based on submissions from Indonesian health authorities, indicated first-dose coverage of 90% in both 2023 and 2024, with second-dose coverage reaching 96% in 2023 and 89% in 2024. These figures align with estimates from Indonesia’s Ministry of Health, which reported approximately 95% first-dose and 90% second-dose coverage for girls aged 11–12 in 2024 [13].

Despite this progress, discrepancies between administrative data and on-the-ground realities have been observed. In Badung District, Bali, where the HPV vaccination program has been implemented since 2016, official records initially reported over 90% coverage in schools. However, a more recent community-based study found lower actual uptake: 82.3% by parental recall and 76.6% verified through immunization records [14]. These findings highlight potential gaps in data accuracy, particularly when administrative coverage is not independently validated. They also underscore the need for more studies exploring urban–rural disparities in vaccine uptake across Indonesia, as existing data may be incomplete, especially regarding differences between urban and rural areas. At present, limited published data from other districts suggest that both coverage inequities and reporting inconsistencies remain important areas for further investigation.

Not receiving vaccination leaves individuals vulnerable not only to acquiring potentially dangerous infections but also to the risk that such infections may become chronic, with far-reaching effects on quality of life, mood, and sexual function. Evidence shows that sexually transmitted infections (STIs), including HPV, disproportionately affect women’s sexual quality of life, leading to challenges such as stigma, embarrassment, and shame. Many women report strained sexual relationships, diminished desire, and reduced frequency of sexual activity following an HPV diagnosis, with some even discontinuing sexual activity altogether out of concern for transmission. These consequences highlight how the burden of infection extends well beyond physical health to encompass psychosocial and relational dimensions, underscoring the importance of vaccination as a preventive tool not only against disease but also against the profound disruption of well-being and intimate life [15,16].

This study aimed to understand HPV vaccination coverage among schoolgirls in urban and rural areas of North Sumatra, Indonesia, and analyze main factors that affect the coverage. We selected Medan (urban) and Deli Serdang (rural), two districts in the Province of North Sumatra with different economic profiles, to conduct this study. We used mixed-methods approaches to investigate the coverage of the HPV vaccine and the gap between Medan and Deli Serdang, to understand the coverage gap between them and identify factors that could be addressed to improve coverage and hence reduce the burden of cervical cancer.

## 2. Materials and Methods

### 2.1. Design and Study Settings

We adopted a mixed-methods approach to collect both quantitative and qualitative data. Quantitative data on HPV vaccine coverage were collected from administrative data of Medan and Deli Serdang, located in the Province of North Sumatra, to offer a glimpse into the urban and rural context in Indonesia. Medan, as the provincial capital, thrives as a commercial and economic center, benefiting from strong infrastructure and global connections. In 2024, Kota Medan recorded a Gross Regional Domestic Product (GRDP) per capita of approximately IDR 132.57 million (around USD 8159). In comparison, Deli Serdang Regency reported a total GRDP of IDR 151,451.40 billion for a population of 2,048,480, resulting in a per capita GRDP of approximately IDR 73.93 million (or about USD 4550) [17,18].

To understand the enabling factors and barriers behind the coverage and the stakeholder perceptions of the vaccination program, we further collected qualitative data in schools in subdistricts with the highest and lowest coverage in Medan and Deli Serdang, respectively. To capture contrasting contexts, letters requesting permission to conduct interviews were sent to schools selected from areas that fell within the top and bottom deciles of HPV vaccination coverage in 2023. This criterion ensured that this study incorporated perspectives from both well-performing and under-performing sites, enabling exploration of factors that may contribute to coverage gaps.

Specifically, in Deli Serdang, focus group discussions and interviews were conducted in two high-coverage primary schools in the Galang subdistrict (90.9% coverage) and in one school from the Tanjung Rejo subdistrict (73.7% coverage). In Medan, one high (67.3%), one medium (55.4%), and one lower (50.4%) coverage area were chosen for qualitative data collection, which corresponded to a Christian, Islamic, and public school. The question design was informed by the WHO Workbook for Conducting a Situation Analysis of Immunization Programme Performance [19].

### 2.2. Data Collection

#### 2.2.1. Quantitative Data

Quantitative data on HPV vaccination coverage among school-aged girls eligible for the 2023 BIAS program were obtained from the Provincial Health Offices of Medan and Deli Serdang. These data were presented in the form of tables and graphs, disaggregated by district, subdistrict, and Puskesmas (community health centers). The datasets included the number of schools under each Puskesmas, the number of girls targeted for vaccination, and the number who received the vaccine. Additionally, municipal-level data were sourced from publicly available government websites.

The Provincial Health Office routinely collects vaccination data through the ASIK system, which tracks vaccine types administered, target populations, and actual coverage rates. This system includes data on both in-school and out-of-school children and monitors administration of both the first and second doses of the HPV vaccine across all districts. In parallel, district health offices record the number of schools under each Puskesmas’ jurisdiction and monitor vaccine delivery by comparing the number of targeted and vaccinated girls.

#### 2.2.2. Qualitative Data

Data were collected primarily through focus group discussions (FGDs), which were designed to elicit shared perspectives rather than individual survey responses. As such, responses were not recorded at the level of individual participants but were instead captured as collective group viewpoints. FGDs were conducted among guardians and teachers of school-aged girls eligible for the 2023 BIAS HPV vaccination program. In addition, in-depth interviews were carried out with health officers responsible for overseeing the program to provide complementary contextual insights.

Exclusion criteria included the following: (i) non-school-going girls eligible for the HPV vaccine; (ii) refusal to participate by eligible guardians or teachers; (iii) parents without daughters; and (iv) parents whose children were either not yet in Grade 5 or had already advanced to middle school, high school, or college.

**Figure 1:**
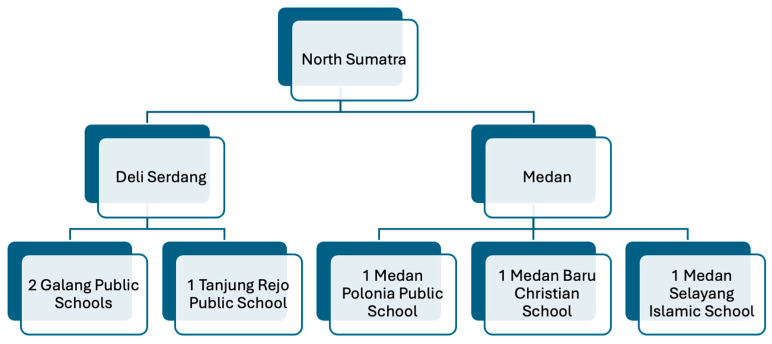
Distribution of schools interviewed in the Deli Serdang and Medan districts, their respective sub-districts, and affiliations.

A total of nine focus group discussions (FGDs) and six individual interviews were conducted as part of this study (see Figure 1 and Table A1). The FGDs were held with parents from both public and private schools in Deli Serdang and Kota Medan, capturing a range of perspectives on childhood vaccination. The interviews were with parents (six focus groups), teachers (six key informant interviews), and local officials (three focus groups) to provide contextual insights that complemented the findings from the FGDs.

The quantitative data provided an objective basis for sampling schools with differing performance levels, while the qualitative methods captured the lived experiences, collective perceptions, and contextual barriers within those settings. Taken together, this mixed-methods approach allowed for a more comprehensive interpretation of both structural and social influences shaping vaccination coverage.

### 2.3. Data Analysis

#### 2.3.1. Quantitative: Vaccination Coverage

The quantitative indicators selected were the total percentage of the target population reached and the total number vaccinated as the numerator and the target number as the denominator, which determined the jurisdiction area of the community health center for selecting schools to interview. The results include coverage rates for both the first and second doses, calculated based on the target population of girls eligible for vaccination that year.

#### 2.3.2. Qualitative: Interviews and Focus Group Discussions

Interview recordings were first manually transcribed verbatim by the research assistant in Bahasa Indonesia, then double translated and verified using both artificial intelligence and native speakers, and reviewed by the primary researcher. The primary author reviewed the transcripts to extract key themes, followed by double coding in NVIVO by the primary author assisted by the qualitative advisor. Thematic analysis was employed based on Braun and Clarke’s 2006 framework to identify key themes from the transcribed data [20].

The socioecological model (SEM) was used as an analytical framework to guide the interpretation and organization of study findings. This model recognizes that health behaviors, such as vaccine uptake, are influenced by multiple levels of influence—ranging from individual beliefs and knowledge to interpersonal relationships, institutional settings, community norms, and broader policy and structural factors [21]. This was performed in this way with the hope of contextualizing barriers and facilitators of HPV vaccine uptake within a broader social and systemic landscape, for identifying intervention points at multiple levels for future program planning.

### 2.4. Ethics

Letters requesting permission to interview were sent to the provincial and district health offices, and permits to conduct the school interviews were also obtained from the residing health office. School and participant recruitment was then performed by sending invitation letters to the school principals, requesting eligible participants. These participants were then gathered at the school and given a participant information leaflet and consent form before the interviews were conducted.

Ethical approval was obtained from Komite Etik Penelitian Kesehatan Universitas Sumatera Utara (Health Research Ethics Committee of Universitas Sumatera Utara) on 7 August 2024, number 1026/KEPK/USU/2024 (Appendix A).

## 3. Results

### 3.1. Coverage Rates of HPV Vaccination by District/School

Across North Sumatra, first-dose coverage across the 33 districts ranged from 17.41% to almost 100% in 2024. The average provincial coverage rate for the first dose of the HPV vaccine in 2023 was 68.63% and 60.27% in 2024. The second follow-up dose of the HPV rate in 2024 from 2023 was 56.13%, reflecting 18,702 missed doses of those eligible.

In 2023, the Deli Serdang district achieved a first-dose HPV vaccination coverage rate of 61.3%, with the same cohort second-dose follow-up in 2024 rising slightly to 63.1%. By comparison, the first-dose coverage for 2024 was 62.1%, indicating consistent vaccination uptake over the two years. In contrast, the Medan district reported significantly lower vaccination rates. In 2023, the first-dose HPV vaccination coverage was 33.4%, and the second-dose follow-up in 2024 dropped to 17.4%. The first-dose coverage in 2024 was 27.2%, reflecting ongoing challenges in vaccine uptake, particularly as HPV vaccinations had only recently been introduced in this district (Table 1).

By districts, Kota Medan covers 954 schools across 21 sub-districts, whereas Deli Serdang covers 1091 schools across 34 sub-districts. The schools chosen from subdistricts in Kota Medan represented coverages of 50.4%, 55.4%, and 67.3% in Medan Polonia, Medan Selayang, and Medan Baru, respectively, and the subdistricts in Deli Serdang represented coverages of 90.9% and 73.7% in Galang and Tanjung Rejo, respectively (Table 2).

In Deli Serdang, most participants chose to vaccinate their children (28/31, 90%), while in Kota Medan, slightly over half did (19/30, 63%). Vaccination rates also varied by school type, with no parents opting in at one private school in Medan and full participation at another. Across public schools in both areas, the rate was 37/41 (90%) (Table A1).

### 3.2. Focus Group Demographics

Table 3 summarizes the demographic characteristics of parents who participated in the FGDs in Deli Serdang (*n* = 31) and Kota Medan (*n* = 30). The variables include relationship, religion, ethnicity, and occupation. The sample included mostly mothers (59/61, 97%); the rest were grandmothers. The most prominent religion was Islam across both districts (42/61, 61%), and the most common ethnicity was Javanese (17/30, 57%) in Deli Serdang, but Batak Toba (11/30, 37%) was followed by Javanese (10/30, 33%) in Kota Medan. A majority of participants in both areas were engaged in informal or home-based work. In Deli Serdang, 83% (25/31) were housewives, while in Kota Medan, there was a higher proportion of other occupations; 59% (17/29) were housewives.

### 3.3. Main Factors Associated with HPV Vaccination Coverage

In Deli Serdang, almost all the parents in the higher coverage Galang subdistrict public schools interviewed elected to vaccinate their children. Although most of the parents attending the focus group discussion in the lower coverage Tanjong Rejo public school chose to vaccinate their girls, it was stated by the teacher and parents that most parents in the school had elected not to vaccinate: ‘Not a single child in (one class) got vaccinated. In (another class), only two kids got the vaccine.’

Most of the parents participating in the focus group discussions in Medan also vaccinated their girls, apart from the Islamic school. According to the transcription data, the parents in the Islamic school had not been aware of what vaccination program was running and hence declined: ‘When we asked what it’s for, the child said they didn’t know. The letter didn’t explain anything either.’

**Figure 2:**
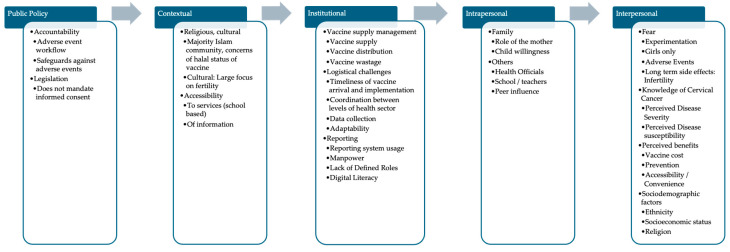
Factors influencing parental decision-making.

We further categorized the factors that affect the HPV vaccine coverage into the SEM to present the qualitative findings (Figure 2, dividing these factors into public policy, contextual, institutional, interpersonal, and intrapersonal [21]).

However, overarching these were trust, information, communication, and education. Across all FGDs amongst parents, trust was found to be adequate in officials. Implicit trust was shown more often in the higher coverage Deli Serdang area. A parent from the Islamic school in Medan commented, ‘But if it’s provided by the government, it must be good, right?’. Implicit trust was shown more often in the higher coverage Deli Serdang area, with parents sharing the sentiment, ‘As far as we know, the health center staff who come are official, and they give clear explanations.’ In Tanjung Rejo, a parent mentioned: ‘We just went along with it. We only asked, “What is this injection for?” and if they explained, we just followed’. In contrast, in Medan a teacher commented the following: ‘Some parents don’t even want to process their child’s birth certificate, let alone vaccinations’.

Most hesitant parents expressed concerns about the vaccination program’s intentions, fearing, “We are also afraid because of rumors saying that the children might be used for medical experiments or that vaccines are being sold.” Others questioned why only girls were vaccinated while boys were not. There were also suspicions about the frequent number of vaccinations—‘Because they were just vaccinated recently, and now it’s HPV again. We didn’t know that they get one shot in grade 5 and another in grade 6.’

Parents wanted to be properly informed before making their decisions. In a higher coverage area, parents received explanations from the Puskesmas. Parents from a lower coverage area reported receiving letters informing them only about the vaccination program and event, but not information about the vaccine, which they had concerns about. They wanted to be properly informed before making their decisions—‘Actually, we don’t mind vaccinations, but at least explain the benefits and risks so we can weigh them properly’.

Most commonly, information was conveyed within and between teachers and parents via WhatsApp groups. However, parents preferred the face-to-face socializations over all other modes of communication because they found the information hard to understand and valued the opportunity to ask questions and voice their concerns. At the two separate schools in the lower coverage Medan area, the following was stated: ‘There was no parents’ meeting. It would be better to have a meeting first so there’s a place to ask questions’, and ‘Notify us first, hold a meeting, and invite us. At the very least, there should be direct communication’.

Both parents and teachers also preferred to obtain their information and have their questions answered by health officials, rather than only from teachers. Parents preferred to have a doctor present: ‘Once the doctors have socialized the information, we’re sure it’s for the best, and we have to accept it’. A teacher made the remark, ‘I hope medical professionals will conduct socialization to better inform parents. Teachers alone are not enough’.

#### 3.3.1. Public Policy

The HPV vaccination program is a nationwide initiative conducted annually under the Joint Decree (SKP) of four ministries. While this legislation does not mandate informed consent, both schools and parents have raised concerns about accountability and continue to request consent taking. The primary concern of parents was accountability in the event of adverse reactions.

The primary concern of parents was accountability in the event of adverse reactions, stating, “We’re afraid something might happen to our daughters”. Schools are similarly apprehensive, ‘The school also doesn’t dare proceed if the parents don’t agree because later, they’ll be held responsible. If anything happens to the child, the school will be blamed’. However, in the higher coverage Deli Serdang area, parents were reassured by the administering staff during the program—‘We informed them that any reactions after vaccination could be promptly treated at the Galang Kota health center’; parent: ‘The Puskesmas also told us to contact them if there were any problems, but nothing happened’.

The procedure in adverse events was clearly communicated to parents and teachers in high coverage areas; teachers from the three highest coverage schools in Galang, Deli Serdang, and the Christian school in Medan commented, ‘The Puskesmas staff are fully prepared (in the case of adverse event)’, ‘The health center staff usually leaves their contact numbers and prepares an allergic reaction kit’, and ‘(Puskesmas staff) even shared their contact numbers so we could reach out and report anytime’.

#### 3.3.2. Contextual Policy

Halal certification was not a significant concern at the Islamic school. Across all schools, mothers demonstrated awareness of cervical cancer and the importance of prevention. Those who elected not to vaccinate their children hesitated to due to a perceived risk that the HPV vaccine could interfere with reproductive health or cause infertility.

#### 3.3.3. Institutional Policy

The immunization program faced several administrative-level challenges, particularly in vaccine supply management, logistics, and reporting systems. Inconsistent vaccine allocation led to shortages, as some districts received fewer doses than expected due to errors in reporting and forecasting. The timeliness of vaccine arrival was also a major issue, with delays caused by slow procurement processes, unpredictable delivery schedules, and coordination gaps between central, provincial, and district health offices. These delays often disrupted planned vaccination activities, which in turn led to lower coverage rates.

Another key challenge has been the ineffective use of the SMILE electronic reporting system. Health workers often fail to enter data, leading to inaccurate records that falsely indicate vaccine surpluses. This, in turn, results in reduced distribution to affected areas. The use of digital reporting systems was hampered by limited digital literacy among some health staff, making it difficult to ensure accurate and timely data entry. Additionally, there were gaps in data collection and coordination between different levels of the health sector. Provincial and district health offices often faced delays in receiving and consolidating reports from frontline workers, impacting their ability to make real-time decisions on vaccine distribution and coverage tracking. These combined challenges resulted in disruptions to immunization planning and execution, ultimately affecting the program’s ability to achieve its coverage targets.

#### 3.3.4. Intrapersonal Policy

The primary challenge in gaining program acceptance was securing approval from fathers, who often refused without providing clear reasons. In some cases, fathers requested safety guarantees from the school or health center before consenting.

Among parents who permitted their children to be vaccinated, mothers typically made the decision independently, with minimal input from spouses, grandparents, children, or peers. While husbands had the authority to veto the decision, any consequences ultimately fell on the mothers.

#### 3.3.5. Interpersonal Policy

Across both districts, knowledge of HPV was scarce. Participants were aware that the HPV vaccine protected against cervical cancer but were not aware of HPV infection being a viral sexually transmitted disease as the primary risk factor for cervical cancer.

Parents who elected to vaccinate were more likely to perceive that their daughters were at risk of cervical cancer. The impression of the parents who refused, from the parents who agreed, was that ‘They think, in the past, without vaccines, everything was fine. So, if our kids aren’t vaccinated, it’s okay’.

Many stakeholders mentioned the cost of the vaccine as a factor in their decision-making. The health officers across the province believed ‘the public would want it if they understood its benefits, especially given its high cost’, and the Deli Serdang district health officers echoed this sentiment: ‘Teachers and parents are willing because they know it’s expensive’. Parents also brought this up in Focus Group Discussions in Deli Serdang—‘Especially since it’s free; others have said they have to pay a lot to get this vaccine’. A teacher in Deli Serdang also used it to convince parents to vaccinate, saying, ‘I reminded them they might regret it later. Especially since, as you mentioned, it turns out the vaccine is expensive’.

## 4. Discussion

This study provides a glimpse into the HPV vaccine coverage and its gap in urban and rural areas of Indonesia, represented by Medan and Deli Serdang, respectively, after the introduction in 2016, and in-depth qualitative insights into HPV vaccination coverage, barriers, and stakeholder perspectives at the district level to understand the influencing factors of the HPV vaccine coverage. This is an area that has been relatively underexplored compared to the predominantly quantitative national or provincial-level studies conducted in Indonesia. By employing a mixed-methods approach that integrates both qualitative and quantitative data, the research offers a more comprehensive understanding of the complex factors influencing HPV vaccination uptake.

The current study found a lower HPV vaccine coverage in Kota Medan, an urban district with a more developed economic profile, than that of Deli Serdang, a more agriculture- and industry-focused economic profile. This is quite counterintuitive, as most of the previous studies have found the HPV vaccine coverage is usually higher in developed or urban areas compared with rural areas, as parents in developed areas always have better access, knowledge, and acceptance of the HPV vaccine [22,23,24]. However, the decision-making process for parents is shaped by a complex interplay of individual, community, and systemic factors, with communication playing a central role. The higher coverage in Deli Serdang, despite being less economically developed, might be attributed to better communications before and during vaccination.

High-coverage schools in Galang, Deli Serdang, benefited from face-to-face socialization sessions that fostered direct dialogue, community engagement, and trust, whereas schools in Medan relied more heavily on written information, which conveyed knowledge but lacked relational interaction [24]. These differences highlight how communication strategies, supported by school–community linkages and outreach infrastructure, can shape parental confidence in vaccination. At the same time, higher internet penetration and economic growth in Medan may have increased exposure to online misinformation, contributing to hesitancy, while supply delays further constrained coverage [25,26]. Together, these findings suggest that both effective community engagement and reliable infrastructure are critical to sustaining high vaccination uptake.

The qualitative findings further revealed several key factors that affected HPV vaccine coverage. First, gaps in information and communication left parents ill-equipped to consent: low-coverage areas relied on terse written notices, whereas high-coverage schools held face-to-face sessions with Puskesmas staff that built trust, clarified benefits and risks, and dispelled rumors. Second, administrative barriers—including inconsistent vaccine allocation and supply management, procurement and delivery delays, coordination gaps between central, provincial, and district health offices, and underuse of the SMILE electronic reporting system due to limited digital literacy—disrupted planning, forecasting, and timely distribution of doses. Third, policy ambiguity and accountability concerns around informed consent and adverse events compounded these challenges, as both parents and schools feared liability without clear procedural safeguards. Finally, sociocultural and interpersonal dynamics—such as paternal veto power, misconceptions about infertility or medical experimentation, low awareness of HPV’s sexual transmission, and perceived vaccine cost despite its free provision—further constrained parental willingness even when maternal support for cervical cancer prevention was strong.

It is also quite surprising to find that parents interviewed in this study reported that peer and religious influence did not significantly impact their decision-making. Previous findings often imply religious belief might be one important factor to affect HPV vaccine coverage [27,28]. For example, one study in Malaysia, a similarly religious country, found the key barrier to the uptake of the HPV vaccine was the belief that the HPV vaccine might promote premarital sexual activity [28]. However, we found during interviews in the current study that parents attributed the low uptake of HPV vaccinations not to religious objections but primarily to a lack of dissemination of information. As such, the findings reflect more on communication gaps than on broader religious or institutional stances within the Islamic school community.

However, this study’s findings should be interpreted with caution due to several limitations. First, we only selected two districts in Indonesia to conduct this study, and the interviews were further constrained to several selected schools. The results could not represent the coverage gap between urban and rural areas in Indonesia, as we only aim to provide a glimpse of the gap and possible influencing factors. Second, there is potential for selection bias due to the purposive selection of study sites, schools, and participants who volunteered to take part. Third, the inclusion of only one Islamic school and one Christian school limits the generalizability of findings related to religious school contexts, particularly since the perspectives shared may not represent those of the broader religious education communities. Although one Islamic school was included in the interviews, its views should not be considered representative of all Islamic schools.

## 5. Conclusions

In conclusion, improving HPV vaccine uptake in Indonesia requires coordinated strategies across all levels of the socioecological model, especially in light of the stark disparities between districts. At the individual and interpersonal level, parents expressed a strong preference for clear, face-to-face communication with trusted sources—particularly healthcare workers and teachers—demonstrating that personal dialogue is key to addressing concerns and building vaccine confidence. At the community and institutional level, both supply- and demand-side challenges were observed. While areas like Deli Serdang benefited from proactive school–Puskesmas collaboration, coverage in urban Kota Medan remained substantially lower, driven by weaker outreach, miscommunication, and inconsistent implementation. At the systems level, administrative staff emphasized the need for improved technological infrastructure and training to support data management and coordination. Collectively, these findings reveal that increasing HPV vaccine uptake requires more than just policy rollout—it demands localized, trust-based engagement, stronger logistics, and administrative readiness tailored to the specific needs of low-performing areas like Kota Medan.

## Figures and Tables

**Table 1:** North Sumatra districts Deli Serdang and Kota Medan coverage data for HPV dose 1 (2023 and 2024) and HPV dose 2 (2024).

District	HPV First Dose 2023		HPV First Dose 2024		HPV Second Dose 2024	
	Target	Coverage	Target	Coverage	Target	Coverage
Deli Serdang	20,059	61.28%	17,465	63.13%	20,254	62.09%
Kota Medan	16,446	33.36%	18,429	17.41%	16,358	27.20%

**Table 2:** Geographic area and vaccination coverage rates of sample sites across sub-districts.

District	Subdistrict	Number of Schools	Target Number	Number Vaccinated	Coverage Rate (%)
Deli Serdang	Galang	31	844	767	90.9
	Tanjung Rejo	62	2416	1781	73.7
Kota Medan	Medan Selayang	34	536	297	55.4
	Medan Baru	28	318	214	67.3
	Medan Polonia	23	381	192	50.4

**Table 3:** Demographics of parents interviewed in the Deli Serdang and Medan districts.

	Relationship		Religion			Ethnicity			Occupation			
	Mother	Grandmother	Islam	Protestant	Other	Java	Batak Toba	Other	Housewife	Teacher	Entrepreneur	Other
Deli Serdang (*n* = 31)	30	1	23	8	0	17	4	10	25	3	1	2
Kota Medan (*n* = 30)	29	1	19	6	5	10	11	9	17	4	5	4

## Abbreviations

The following abbreviations are used in this manuscript:

HPV	Human papillomavirus
CIN	Cervical intraepithelial neoplasia
MICs	Middle-income countries
YLL	Years of life lost
WHO	World Health Organisation
GDRP	Gross Domestic Regional Product
JKN	Jaminan Kesehatan Nasional
BIAS	Bulan Imunisasi Anak Sekolah
UNDP	United Nations Development Programme
SMILE	Sistem Monitoring Informasi Logistik secara Elektronik
ASIK	Sehat IndonesiaKu
Puskesmas	Pusat Kesehatan Masyarakat
ANCCA	Asian National Cancer Centers Alliance
FGDs	Focus group discussions
KIIs	Key informant interviews
SEM	Socioecological model

## Appendix A

**Table A1:** Affiliation and vaccination status data of sample sites.

School ID	District	School Affiliation	Parents Interviewed	Vaccinated	
				Yes	No
H1	Deli Serdang	Public	10	10	0
H2	Deli Serdang	Public	11	11	0
L3	Deli Serdang	Public	10	7	3
L4	Kota Medan	Islamic	10	0	10
L5	Kota Medan	Christian	10	10	0
L6	Kota Medan	Public	10	9	1
